# Epigenetic regulation of immune checkpoint signaling by microbial metabolites: mechanisms, therapeutic potential, and future perspectives

**DOI:** 10.3389/fimmu.2026.1970192

**Published:** 2026-10-01

**Authors:** Asiya Nazir, Rafia Nazir, Sarika Sharma, Touseef Amna, Zienab Fawzy Reiad Ahmed

**Affiliations:** 1 College of Health Sciences, Abu Dhabi University, Abu Dhabi, United Arab Emirates; 2 Division of Drug Discovery & Development Research, Cancer Research Institute, Office of Research and Sponsored Programs, Abu Dhabi University, Abu Dhabi, United Arab Emirates; 3 Department of Anatomy, Skims Medical College and Hospital, Jammu and Kashmir, Srinagar, Kashmir, India; 4 Department of Sponsored Research, Division of Research Connect Lovely Professional University, Phagwara Punjab, India; 5 Department of Biology, Faculty of Science, Al-Baha University, Al-Baha, Saudi Arabia; 6 Department of Integrative Agriculture (INAG), College of Agriculture and Veterinary Medicine (CAVM), United Arab Emirates University (UAEU), Al Ain, United Arab Emirates

**Keywords:** gut microbiome, host–microbiota interactions, immune checkpoint signaling, immunometabolism, metabolite–immune checkpoint axis, microbial metabolites, personalized microbial therapeutics, tumor microenvironment

## Abstract

The gut microbiome has emerged as an important regulator of host immunity through the production of diverse bioactive metabolites. The gut microbiome has emerged as an important regulator of host immunity through the production of diverse bioactive metabolites. Microbiota-derived metabolites influence immune cell function and contribute to the maintenance of immune homeostasis, serving as key molecular mediators of host–microbiota communication. Importantly, while evidence is emerging that these metabolites affect immune checkpoint signaling, less is known about the molecular mechanisms behind these interactions. Recent research demonstrated that SCFAs, secondary bile acids, indoles derived from tryptophan, inosine, polyamines and other microbial metabolites affect the immune checkpoints through epigenetics, metabolism, receptor signaling, and intracellular pathway modulation. These interactions affect the expression and functioning of key immune checkpoint molecules such as PD-1, PD-L1, CTLA-4, LAG-3, TIGIT, TIM-3 and VISTA. In this review, we summarize current knowledge regarding the Metabolite–Immune Checkpoint Axis, stressing the role of metabolites derived from microbiota in the regulation of immune checkpoints. We elaborate on the molecular mechanisms of these interactions and their significance in the context of cancer, autoimmune disorders, chronic inflammatory disorders, and infectious diseases. We also provide insights regarding the developing therapeutic strategies based on microbiome-modulated therapy. Finally, we highlight key knowledge gaps and future research directions required for the clinical translation of microbiome-based personalized therapies.

## 1 Introduction

One of the most complex microbial ecosystems resides in the human gastrointestinal tract, in which there are trillions of bacteria, archaea, fungi, viruses, and several other microorganisms, together known as gut microbiota. Rather than being passive commensals, these microorganisms have coevolved with the host for the acquisition of a continuously changing and mutually advantageous relationship, which contributes to maintaining homeostasis in the body. Besides their already-recognized functions in nutrient metabolism, vitamin production, and prevention of pathogen invasion, gut microbiota plays an essential role in immunological processes. Numerous studies show that constant communication between microbes and an immune system of the host organism is necessary for the preservation of immune tolerance and the ability to react to dangerous microorganisms (1). Communication between the host and microbiota involves a network of interactions at a molecular level including, microbial-associated molecular patterns (MAMPs), PRRs, microbial metabolites, and immune signaling pathways. The PRRs, such as Toll-like receptor (TLRs), NOD-like receptors (NLRs), and C-type lectin receptors present on the gut epithelial cells and innate immune cells, play a key role in recognizing microbial-associated molecular patterns (MAMPs) and starting signaling cascades that lead to cytokine secretion, antigen presentation, and maturation of the immune system (2). The communication between host and microbiota creates fine balance between immune activation and tolerance under healthy conditions preventing the overreaction of the immune system against beneficial microbes while still preparing for the pathogenic invasion (3).

The largest immune compartment of the human body is located within the intestinal mucosa and involves a constant interaction between epithelial cells, macrophages, dendritic cells, innate lymphoid cells, and both B and T lymphocytes. The processes being elaborated in the intestinal mucosa are responsible for the activation of regulatory T cells (Tregs), maintaining IgA production, and performing various functions of the immune system (4, 5). Some commensal microorganisms have beneficial effects and lead to the stimulation of immunological tolerance and anti-inflammatory pathways, while dysbiosis creates an environment in which regulatory pathways are disrupted. Hence, the healthy state of the gut microbiota is preserved (6).

Although the composition of the gut microbiota provides valuable information about microbial diversity, it does not fully capture the functional contribution of the microbiome to host physiology. Consequently, research has increasingly shifted from characterizing microbial community composition toward identifying bioactive metabolites produced by these communities, as these metabolites serve as important mediators of host–microbiota interactions (2). These low-molecular-weight compounds are generated through microbial metabolism of dietary or endogenous substrates and can influence host cellular functions, metabolism, and immune responses. Thus, microbial metabolites provide a functional link between the gut microbiota and host immunity and may have important implications for precision medicine (7). Short-chain fatty acids (SCFAs) represent the most studied metabolites, among which acetate, propionate, and butyrate have been extensively explored due to their diverse immune-modulating properties. SCFAs are generated through the fermentation of dietary fibers by bacteria and exert their immune effect by means of several G protein-coupled receptor activations and histone deacetylases (HDACs) suppression involved in transcription and cytokines synthesis and immune cell differentiation. Apart from SCFAs, other microbiota-produced compounds like secondary bile acids, tryptophan-derived indole metabolites, inosine, polyamines, and others act as important factors influencing both innate and adaptive immunity due to their capacity to bind to various receptors, including aryl hydrocarbon receptor (AhR), farnesoid X receptor (FXR), and TGR5 receptor (8). These metabolites act altogether by influencing the epithelial barrier integrity, facilitating differentiations of regulatory T-cells, and regulating macrophage polarization and immune tolerance.

Immune checkpoint molecules play an essential role in overseeing immune homeostasis, allowing a fine equilibrium to be maintained between a successful defense in the body and immune tolerance. These receptor molecules are present in a variety of immune cells and act as the brake mechanism for modulating excessive immune activation, thus avoiding damage to the tissue and preserving the self-tolerance after antigen activation. One of the most studied representatives of immune checkpoint molecules is programmed cell death 1 (PD-1), programmed death-ligand 1 (PD-L1), cytotoxic T-lymphocyte-associated protein 4 (CTLA-4), LAG-3, TIM-3, TIGIT, and VISTA. These receptors interact with their ligands to govern T-cell activation, proliferation, cytokine secretion, and action, as well as to affect dendritic cells, macrophages, NK cells, and regulatory T cells. In normal circumstances, immune checkpoint signaling is crucial to maintaining peripheral tolerance and preventing any autoimmune disorder; however, disturbances of these pathways lead to chronic inflammation, immune failure, long-term infections, autoimmune disease, and tumor immune escapement (9–11).

Recent developments in immunology have significantly broadened the understanding of immune checkpoint molecules, which were initially known for their use in cancer immunotherapy. New discoveries show that checkpoint signaling is affected by many environmental and metabolic factors present in the tissue microenvironment, and these factors can come from the gut with the help of certain organisms (12). According to the newly formed belief, checkpoint activity depends not only on the genome of the host and inflammatory mediators, but also on the antimicrobial products that contribute to the regulation of the path of cellular signals, metabolism, and the development of epigenetics. Hence, the analysis of the interaction between the substances produced by the microorganisms and the immune checkpoint signaling has become a popular research subject that can help to investigate the ways of immunoregulation and to provide the material for the creation of new therapeutic measures based on microbiota (13).

Microbiota-derived metabolites are increasingly recognized as important regulators of immune-cell differentiation, metabolism, and inflammatory signaling, while immune checkpoints are well-established regulators of immune activation and tolerance. However, the molecular mechanisms directly linking specific microbial metabolites to immune checkpoint expression and function remain incompletely understood. In particular, the relative contributions of receptor-mediated signaling, epigenetic regulation, and metabolic reprogramming require further clarification. Integrating these areas is therefore essential for understanding how microbial metabolism shapes checkpoint-mediated immunity and for identifying new opportunities for microbiome-based precision immunotherapy.

Existing reviews have largely examined gut microbiome composition, microbial metabolites, immunometabolism, or immune checkpoint biology as separate or broadly interconnected areas. However, comparatively limited attention has been given to systematically integrating the molecular mechanisms through which specific microbiota-derived metabolites influence immune checkpoint expression and function. The present review addresses this gap by focusing specifically on metabolite–receptor interactions, intracellular signaling pathways, epigenetic regulation, and metabolic reprogramming that connect microbial metabolism with immune checkpoint control.

Accordingly, this review introduces the Metabolite–Immune Checkpoint Axis as a unifying conceptual framework that links specific microbiota-derived metabolites with host receptors, intracellular and epigenetic signaling mechanisms, immune-cell responses, and ultimately immune checkpoint expression and function (**Figure 1**). Unlike the broader microbiome–immune checkpoint relationship, which encompasses associations between microbial community composition and immune checkpoint responses, the Metabolite–Immune Checkpoint Axis specifically focuses on the mechanistic links through which defined microbiota-derived metabolites influence checkpoint expression and function We summarize the current knowledge on the mechanisms of the effect of microbial metabolites on the immune checkpoints pathways, and analyze their potential role in the immune-related diseases and possible applications of microbiome-based therapies for personalized treatment based on microbial products.

**Figure 1:**
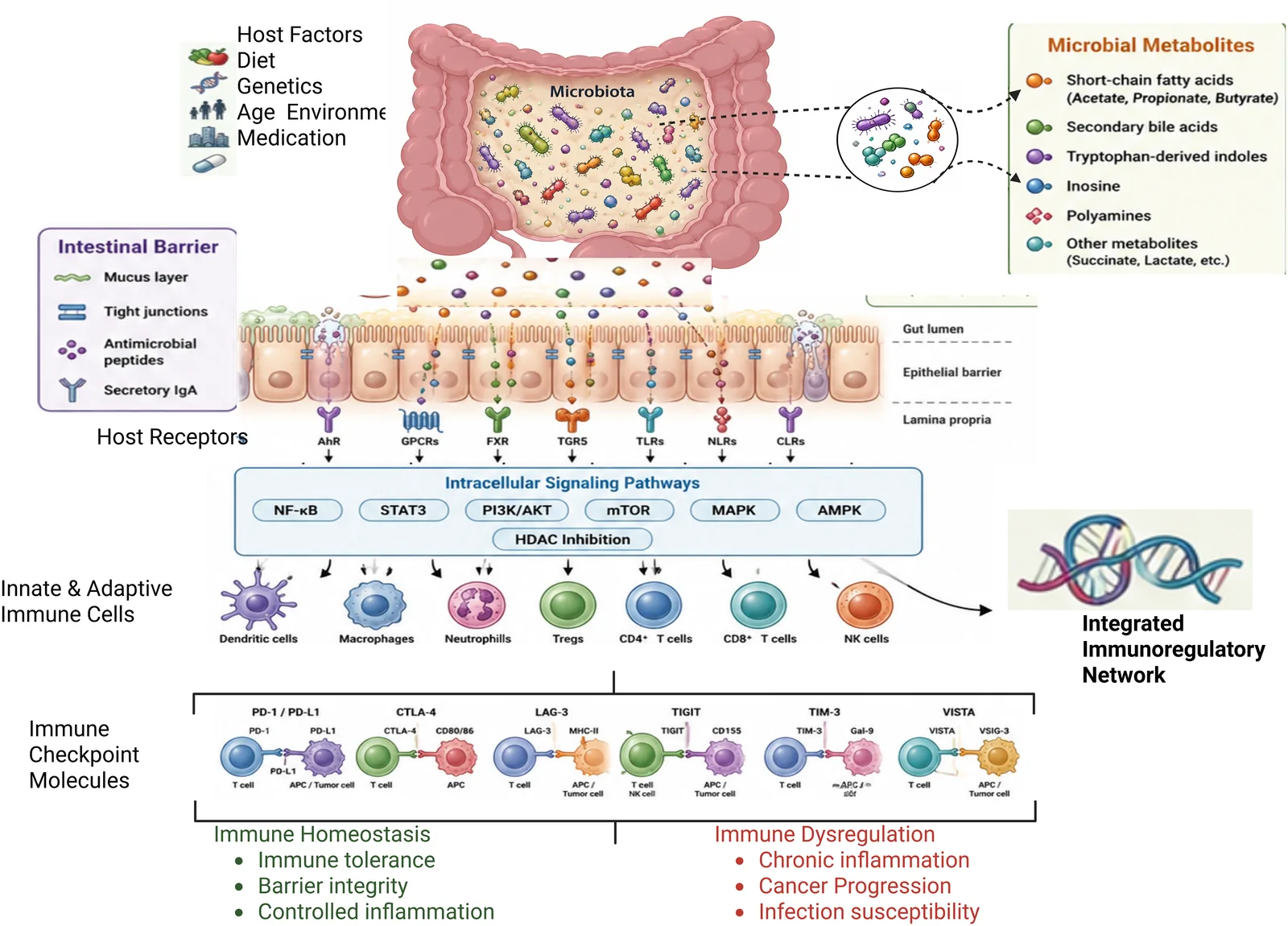
Conceptual framework illustrating the host–microbiota–metabolite–immune checkpoint axis. Host factors influence the composition and metabolic activity of the gut microbiota, leading to the production of bioactive microbial metabolites. These metabolites interact with host receptors and intracellular signaling pathways in intestinal epithelial and immune cells, thereby regulating immune cell function and immune checkpoint signaling. This integrated network determines immune homeostasis or immune dysregulation and provides a mechanistic basis for the development of personalized microbial therapeutics. (Created in BioRender. Nazir, A. (2026) https://BioRender.com/i6ws9bw).

## 2 The gut microbiome as a central regulator of host immunity

The gut microbiome plays an important role in the development and regulation of host immunity through continuous interactions with the intestinal immune system. These interactions influence both innate and adaptive immune responses and contribute to immune homeostasis through direct host–microbiota interactions and microbiota-derived metabolites. This section discusses host–microbiota interactions, immune homeostasis, and dysbiosis as the biological basis for understanding how microbiota-derived metabolites may influence immune checkpoint regulation.

### 2.1 Host–microbiota immune interactions

The gut is one of the largest and most immunologically active sites of interaction between hosts and the environment that is home to microbes and other microorganisms. This association has developed over millions of years and transformed into an important part of the immune system rather than group of microorganisms. Thus, the relationship between the host and microbiota is essential for immunity development and maintenance of the intestine’s homeostasis (11, 14). The connection entails continuous interaction between the microbial community and intestinal epithelial and immune cells, which means that this process is also called host–microbiota crosstalk (11). It is worth noting that any failure in the system can contribute to the development of various chronic diseases (15).

The interaction between the host and its microbiota starts by identifying the various MAMPs, which include lipopolysaccharides, teichoic acids, flagellin, lipoteichoic acid, and microbial nucleic acids. The PRRs exist in various cells such as the epithelial cells and innate immune cells. Some of these receptors are TLRs, NLRs, and CLR that all have many unique ligands and mechanisms for sending signals (16). PRR activation can induce either protective or inflammatory immune responses depending on the receptor involved, microbial ligand, responding cell type, and signaling context. Controlled PRR signaling contributes to intestinal homeostasis and host defense, whereas excessive or dysregulated activation may promote inflammation. Rather than acting as sensors of infection, PRRs actively survey the environment of the intestines and understand the signals in the surroundings of the intestines so that they can engage the processes of tissue repair, maintaining the integrity of the barrier, and achieving immune tolerance. Hence, PRR signaling can be explained as a means through which the host regulates its interactions with the pathogens present in its environment (17).

### 2.2 Maintenance of immune homeostasis

The intestinal immune system must operate under strict conditions that allow controlled contact with innumerable commensal microorganisms and food antigens at the same time being able to respond adequately to pathogenic invaders. This is known as the phenomenon of immune homeostasis that can be achieved through the collaboration of the gut microbiome, the gut epithelial barrier, and the immune system. Instead of being thought of as a static barrier, the intestinal mucosa is a dynamic immunological platform, the interface where the microbial signals influence the integrity of the epithelial barrier, differentiation of immune cells, and cytokine production (18). Failure to maintain the equilibrium may lead to unnecessary activation of the immune response, chronic inflammation, and the organism’s vulnerability to immune-mediated diseases, which shows us the significance of well-regulated interaction between the host and microbiota (19, 20).

In order to keep and ensure proper functioning of the immune system, it is critical to keep the intestinal barrier intact. This barrier includes tightly interconnected epithelial cells, tight junction proteins, mucus, antimicrobial peptides, and secretory IgA (sIgA) (21). The combination of these barriers protects from pathogens but allows for tolerance to commensal micro-organisms. It should be noted that the barrier provides not only protection but also works together with the immune system, producing cytokines and growth factors for epithelial renewal, leucocyte migration and tissue repair. Thus, it can be concluded that keeping the barrier intact is highly important (22).

The gut microbiota also contributes to immune equilibrium because it affects the differentiation and activity of immune cells that control immune reactions. Microbes inhibit certain immune mechanisms, resulting in the stimulation of T regulatory cells (Tregs), promote the production of cytokines of the “anti-inflammatory” type, such as interleukin-10 (IL-10) and transforming growth factor β (TGF-β), and intensify the synthesis of secretory immunoglobulin A (IgA), which ultimately boosts immune tolerance within the intestines (23). Controlled differentiation of the Th17 population provides an antimicrobial defense that minimizes tissue damage. The presence of an adequate balance between the Treg and Th17 responses is essential for maintaining unperturbed immune homeostasis in the gastrointestinal system. Any disturbance in this balance can lead to inflammatory bowel disease, allergies, autoimmune diseases, and cancer. These data prove that immune homeostasis requires action not only upon proper microbial recognition but also constant regulation of the epithelial functions, cytokine systems, and adaptive immunity, which is determined by microbiota (24).

### 2.3 Dysbiosis and immune dysregulation

To maintain immune homeostasis, it is essential to maintain a stable and diverse gut microbiota. Disturbances to this microbiota have the potential to cause dysbiosis, which is characterized by loss of microbial diversity, loss of beneficial commensal bacteria, and overgrowth of harmful pathogens. Such changes can disturb interactions between the microbiota and the immune system, leading to impaired epithelial barrier function, abnormal immune activation, and chronic inflammation. Dysbiosis is now believed to be a common feature of a number of chronic diseases, bringing to light the importance of a balanced microbiome system for the health of both the gut and the immune system (6, 25).

The most significant ramification of dysbiosis is the destabilization of the intestinal epithelial barrier which, in turn, results in increased intestinal permeability, termed as ‘leaky gut.’ The dysfunction of the barrier permits the entry of microbial-associated molecular patterns, endotoxins, and other organisms’ components into deeper tissues and the systemic circulation, thus kick-starting the series of events that activate the innate immunity (26). The continuous activation of pattern recognition receptor is responsible for increased secretion of pro-inflammatory cytokines, such as TNF-α, IL-6, IL-1β, which in turn creates a chronic inflammatory environment. The constant inflammation adversely affects the process of epithelial recovery thus reinforcing the cycle of impaired barrier function and dysfunctional immune response (27).

Not only does dysbiosis lead to inflammation in your gut, but it also affects the entire immune system in your body. Changes in microbiota composition are considered risk factors for a range of diseases: inflammatory bowel disease, rheumatoid arthritis, multiple sclerosis, systemic lupus erythematosus, obesity, type 2 diabetes, and cancer. In cancer, dysbiosis promotes inflammation and modifies the tumor environment, thus influencing immune cell influx, cytokine production, and immune checkpoint expression (28). Although direct interactions between the host and the microbiome are of importance, recent studies demonstrate that many of the mentioned immune effects are based on the changes of microbial metabolites produced by the gut microbiome. Thus, changes in the production of metabolites can be considered one of the crucial mechanisms leading to dysbiosis and malfunctioning of the immune system (29). The major cellular and molecular components involved in host–microbiota interactions and their contributions to immune regulation are summarized in **Table 1**.

**Table 1:** Major cellular and molecular components of host–microbiota interactions and their immunological functions.

Component	Primary function	Contribution to immune regulation	Mechanistic link to immune checkpoints	Representative references
Intestinal epithelial cells	Physical and immunological barrier; cytokine and antimicrobial peptide secretion	Maintain barrier integrity and coordinate mucosal immunity	Barrier/inflammatory signaling → T cells and other mucosal immune cells → may indirectly influence checkpoint expression through the local immune environment	(30, 31)
Pattern recognition receptors (TLRs, NLRs, CLRs)	Recognition of microbial-associated molecular patterns (MAMPs)	Initiate innate immune signaling and immune surveillance	PRR-mediated signaling (e.g., NF-κB/MAPK) → innate immune cells → cytokine-dependent modulation of checkpoint pathways	(16, 17)
Goblet cells	Mucus secretion	Prevent microbial translocation and support mucosal protection	Maintenance of barrier integrity → limits inflammatory immune activation → **indirect relationship with checkpoint regulation**	(30)
Paneth cells	Secretion of α-defensins, lysozyme and antimicrobial peptides	Control microbial composition and intestinal defense	Regulation of microbial composition and barrier defense → alters immune signaling → **indirect relationship with checkpoint regulation**	(31)
Dendritic cells	Antigen sampling and presentation	Regulate T-cell activation and immune tolerance	Antigen presentation/costimulatory signaling → T-cell activation and tolerance → checkpoint pathways including PD-1 and CTLA-4	(32)
Macrophages	Phagocytosis and cytokine production	Maintain tissue homeostasis and resolve inflammation	Cytokine and inflammatory signaling → T-cell responses and macrophage polarization → may influence PD-1/PD-L1 signaling	(33)
Regulatory T (Treg) cells	Immune suppression	Maintain immune tolerance and prevent excessive inflammation	Immunoregulatory signaling → suppression of effector T-cell responses → associated with checkpoint-mediated immune tolerance, particularly CTLA-4/PD-1	(34)
Th17 cells	Mucosal antimicrobial defense	Protect against pathogens while maintaining immune balance	Cytokine-mediated inflammatory signaling → T-cell functional state → may indirectly affect checkpoint expression	(24, 34)
Secretory IgA	Neutralization of pathogens	Preserve host–microbiota homeostasis without excessive inflammation	Regulation of microbial exposure and mucosal homeostasis → **indirect relationship with checkpoint regulation**	(23, 35)
Gut microbiota-derived metabolites	Intercellular signaling molecules	Regulate immune cell differentiation and immune homeostasis	Receptor-mediated, metabolic and epigenetic signaling → T cells/Tregs/macrophages → modulation of immune checkpoint pathways	(2, 8)

Bold text highlights the nature of the mechanistic relationship with immune checkpoint regulation.

## 3 Microbial metabolites: molecular mediators of host–microbiota crosstalk

The metabolites produced by microbiota act as the functional connection between the gut microbiome and the host immune system. This translates the activity of bacteria into various immune responses. These metabolites, which are a result of the interaction of various substrates either taken from food or produced in our body by metabolism, can maintain immune homeostasis by controlling epithelial integrity, immune cell activity, and intracellular signaling pathways. This section discusses the main types of microbial metabolites available and their mechanisms of action. This section discusses the main types of microbial metabolites available and their mechanisms of action (**Figure 2**).

**Figure 2:**
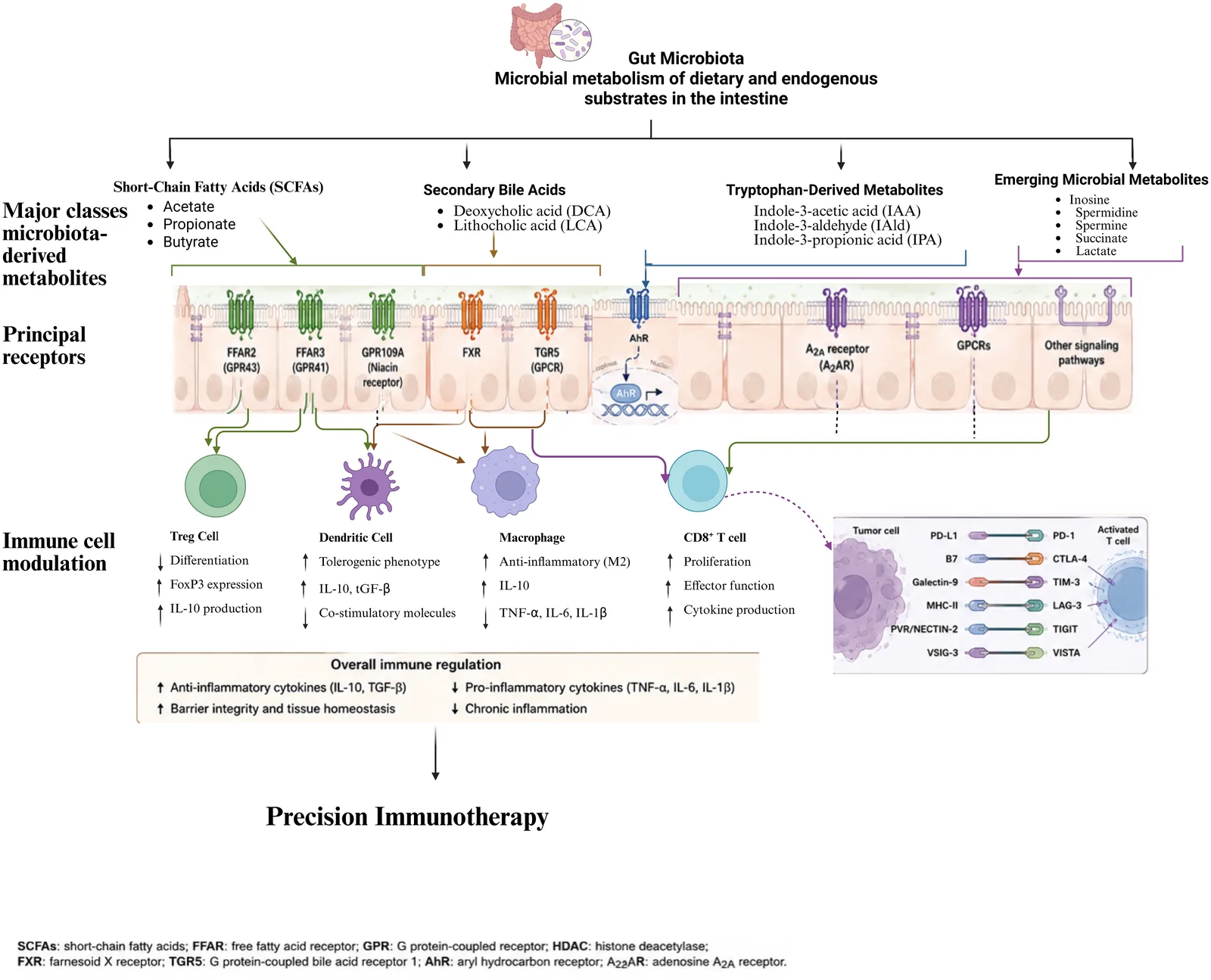
Major classes of microbiota-derived metabolites and their principal host receptors involved in immune regulation. Gut microbial metabolism generates diverse bioactive metabolites, including short-chain fatty acids (SCFAs), secondary bile acids, tryptophan-derived metabolites, and emerging metabolites such as inosine, polyamines, succinate, and lactate. These metabolites interact with distinct host receptors, including FFAR2/3, GPR109A, FXR, TGR5, AhR, and A_2_AR, to regulate immune cell function, epithelial barrier integrity, and inflammatory signaling. Collectively, these interactions contribute to immune homeostasis and provide the mechanistic foundation for microbial modulation of immune checkpoint signaling and precision immunotherapy (Created in BioRender. Nazir, A. (2026) https://BioRender.com/yagxmxy).

### 3.1 Short-chain fatty acids

Short-chain fatty acids (SCFAs) are the most well-known and researched metabolite by microbiota, and thus, they play an important role in communication between the host and microbiota (36, 37). These molecules are produced as a result of anaerobic fermentation of non-digestible dietary fibers and resistant starch by various commensal bacteria inside the colon. The proportion of acetate, propionate, and butyrate represents over 95% of the total amount of SCFAs present in the intestinal lumen. Acetate is produced by bacteria belonging to the genera Bifidobacterium and Akkermansia, while propionate is formed by members of the species Bacteroides and Veillonella. Butyrate is formed by Firmicutes bacteria, such as *Faecalibacterium prausnitzii*, Roseburia spp., and *Eubacterium rectale*.

Dietary composition, microbial diversity, and intestinal environmental conditions play a crucial role in determining the production and relative abundance of SCFAs, thus showing how nutrition interacts with gut microbiota and the body (36). SCFAs have not only metabolic end products but also important signaling molecules regulating the immune responses, intestinal homeostasis, and susceptibility to different diseases by regulating various signaling pathways in the body and thus giving an important link between microbial metabolism and immune regulation (36–38).

The immunomodulatory action of SCFAs occurs through the activation of G protein-coupled receptors (GPCRs) like free fatty acid receptor 2 (FFAR2/GPR43)), FFAR3 (GPR41), and GPR109A and inhibition of histone deacetylases. The activation of those receptors in immune and intestinal epithelial cells and that contributes to the modulation of different signa.ing pathways that are important for maintaining immune homeostasis (39).

GPR43 and GPR41 can be found in neutrophils, macrophages, dendritic cells, and intestinal epithelial cells, where they are responsible for processes such as chemotaxis and the release of various cytokines. Specifically, GPR109A, which binds closely with butyrate, is essential for activating anti-inflammatory signaling pathways, maintaining the integrity of the epithelial barrier, and enabling mucosal immune tolerance. Butyrate and propionate not only act through receptor-mediated pathways but also serve as endogenous HDAC inhibitors, which means they bring about epigenetic changes in chromatin accessibility and regulate the expression of genes that play various roles in immune modulation, cell differentiation, and inflammation processes (40). These processes show how SCFA connects microbial metabolism with host signaling mechanisms thus linking intestinal fermentation of dietary fibers with immune modulation (39).

SCFAs have wide-ranging effects in immunity by regulating the differentiation and activity of innate and adaptive immunity cells. Among those SCFAs butyrate is the most potent immune-modulator that promotes Treg cells differentiation and stability through the modulation of FoxP3 (39, 41). SCFAs are also capable of inducing the tolerogenic phenotype in dendritic cells and promoting anti-inflammatory macrophage polarization leading to increased IL-10 and decreased release of pro-inflammatory cytokines (TNF-α, IL-6, and IL-1β). In addition, SCFAs contribute to the intestinal barrier integrity by enhancing tight junction proteins expression and epithelial repair, which limits the translocation of bacteria and preserves mucosal immune homeostasis (41). Collectively, all these processes lead to the fact that SCFAs can be considered the key regulators of immune balance that form the background for their influence on the immune checkpoint signaling (42) (Ghosh et al., 2026).

While SCFAs have long been known for their roles in immune balance, recent findings suggest that SCFAs also have a role in immune checkpoint signaling, thus affecting anti-cancer immunity and response to immune checkpoint inhibitors. Of the SCFAs, butyrate has been the most studied because of its physiological property of impacting the expression and functioning of immune checkpoint proteins through receptor- and epigenetic-dependent ways. Butyrate has been reported to inhibit HDAC activity, which results in changes in the transcriptional activity of PD-1 and PD-L1 genes, as well strengthening activation, multiplication and functioning of CD8^+^ T lymphocytes (43). It can help improve immune monitoring and support effective anti-cancer responses. Furthermore, differences in the levels of SCFAs and variations in microbiome composition are associated with the degree of response to immunotherapy, which means that microbial metabolites can be potential biomarkers of treatment efficacy (44).

The exact mechanisms responsible are not completely known but current information shows that SCFAs might have a key role in the modulation of immune checkpoint signaling requiring further investigation in microbiome-based precision immunotherapy (37).

### 3.2 Secondary bile acids

Microbial biotransformation of primary bile acids leads to the formation of secondary bile acids, which are an important kind of metabolite produced by microbiota that has signaling properties too. Secondary bile acids are produced via bacterial deconjugation and 7α-dehydroxylation, in contrast with SCFA, which come from dietary fiber fermentation. The main secondary bile acids, deoxycholic acid (DCA) and lithocholic acid (LCA), have more than just their classical role in fat digestion, and are also capable of regulating host physiological and immune functions (45, 46). The important mechanism of their biological action is considered to be activation of the farnesoid X receptor (FXR) and G protein-coupled bile acid receptor 1 (TGR5), which are highly disseminated on intestinal epithelium and immune cells, responsible for mediating their effects. The activation and stimulation of these receptors are responsible for the functionality of bile acids, function of the epithelial barrier, signaling of inflammation, and metabolic homeostasis, which proves that there is a close connection between the process of bile acids metabolism performed by microbes and host physiology (47).

This becomes obvious due to the available findings confirming that secondary bile acids are capable of carrying out an immune influence by adjusting dendritic cells, macrophages, and T cells with the help of the activation of FXR and TGR5. These metabolites are possible to activate anti-inflammatory processes through cytokine production regulation, maintaining epithelial integrity, and provision of the immune tolerance in the intestinal microenvironment (48). These experiments show the connection between modifications of bile acids metabolism, tumor microenvironment, and immune checkpoint blockade efficiency only representing the tip of the iceberg.

### 3.3 Tryptophan-derived metabolites

Metabolites of tryptophan are generated via microbial digestion of food tryptophan and represent an important group of bioactive substances serving to communicate between host and microbiota. However, unlike SCFAs and secondary bile acids, these metabolites mainly function through AhR activation, a ligand-dependent transcription factor associated with control of immune homeostasis and function of mucosal barrier. The *Lactobacillus, Clostridium, Peptostreptococcus, and Bacteroides* bacteria process tryptophan to form various indole derivatives, including indole-3-acetic acid, indole-3-aldehyde, indole-3-propionic acid, and indole acrylic acid. These metabolites take part in sustaining intestinal homeostasis by controlling epithelial integrity, the production of antimicrobial peptides, and the mucosal immune response (37, 49, 50).

By promoting the activation of AhR by the metabolites of tryptophan synthesized by bacteria, the differentiation and activity of different types of immune cells - including T-regulatory, innate lymphoid, dendritic, and macrophage cells - is modulated, thus resulting in the activation of immune tolerance and the limiting of intense inflammatory reaction. Moreover, the signaling through AhR is able to determine the amount of IL-22 produced, which is essential for the maintenance of the integrity of epithelial barrier, healing of the tissues, and protection of the body from various microorganisms (51). Recent data suggests that products of tryptophan metabolism by intestinal microbiome can also influence the immune tumor microenvironment through alteration of inflammatory signaling pathways and work of immune cells, thus implying their role in the regulation of checkpoint blockade. Although the mechanisms of their action still remain unknown, the AhR pathway has the potential to become one of the most powerful links between microbiome metabolism and precision immunotherapy (52, 53).

### 3.4 Emerging microbial metabolites

Various microbiota-derived metabolites have been identified as significant modifiers of host immunity, and this includes not only SCFAs, bile acids, and tryptophan metabolites. This category includes such compounds as polyamines, inosine, lactate, and succinate that are produced via purine metabolism and amino acids transformation. Even though the above metabolites were studied to a lesser extent, the body of knowledge suggesting their impact on immune response, inflammatory pathways, protection of epithelial cells, and above all, cellular metabolism is growing. These compounds represent important metabolization byproducts that trigger different intracellular signaling pathways and bind to some specific cells, which shows that microbial metabolism is connected with immune regulation of host.

In particular, inosine has been one of the most researched compounds among metabolites in terms of its influence on stimulation of anti-cancer immunity via functioning of adenosine A_2_A receptor. Polyamines such as spermine and spermidine are involved in cell growth and differentiation of immune cells, as well as maintaining intestinal mucosa integrity while succinate and lactate are responsible for inflammatory signaling pathways through regulating macrophage reprogramming, hypoxia-associated pathways (54). Although the details related to the molecular mechanisms behind these substances are not entirely clear yet, accumulating experimental data suggests that they could be involved in immune checkpoint regulation either directly or indirectly using metabolism and epigenetic reprogramming of immune cells. Thus, further studying these new metabolites could give an opportunity to find new pharmacological agents to be used in microbiome-based immunotherapy (55).

## 4 Molecular mechanisms linking microbial metabolites to immune checkpoint signaling

The metabolites produced by microbiota can exert effects on immune checkpoint signaling via various molecular mechanisms that are interlinked, such as receptor signaling, epigenetic regulation, and metabolic reprogramming. These mechanisms affect immune cells in different ways, allowing them to play a significant role in the activity of immune checkpoint receptors, including PD-1, PD-L1, CTLA-4, LAG-3, TIM-3, TIGIT and VISTA. These findings illustrate the various pathways through which microbial metabolism affects antitumor immunity. To provide a systematic mechanistic framework, the following sections examine these interactions along the sequence: microbial metabolite → host receptor or molecular target → intracellular signaling pathway → affected immune cell → checkpoint molecule → biological outcome. This framework also distinguishes direct regulation of checkpoint expression from indirect effects mediated through immune-cell differentiation, cytokine signaling, metabolic reprogramming, or the tumor microenvironment. Importantly, the strength of evidence supporting these interactions varies considerably. These integrated molecular mechanisms are illustrated in **Figure 3**. For several metabolites, current findings demonstrate associations with immune-cell function or responses to checkpoint blockade rather than direct regulation of checkpoint molecules. Therefore, mechanistic conclusions should be interpreted according to the experimental model and the availability of direct molecular evidence. The major microbiota-derived metabolites, their molecular targets, affected immune cells, checkpoint associations, mechanisms, and current levels of supporting evidence are summarized in **Table 2**.

**Figure 3:**
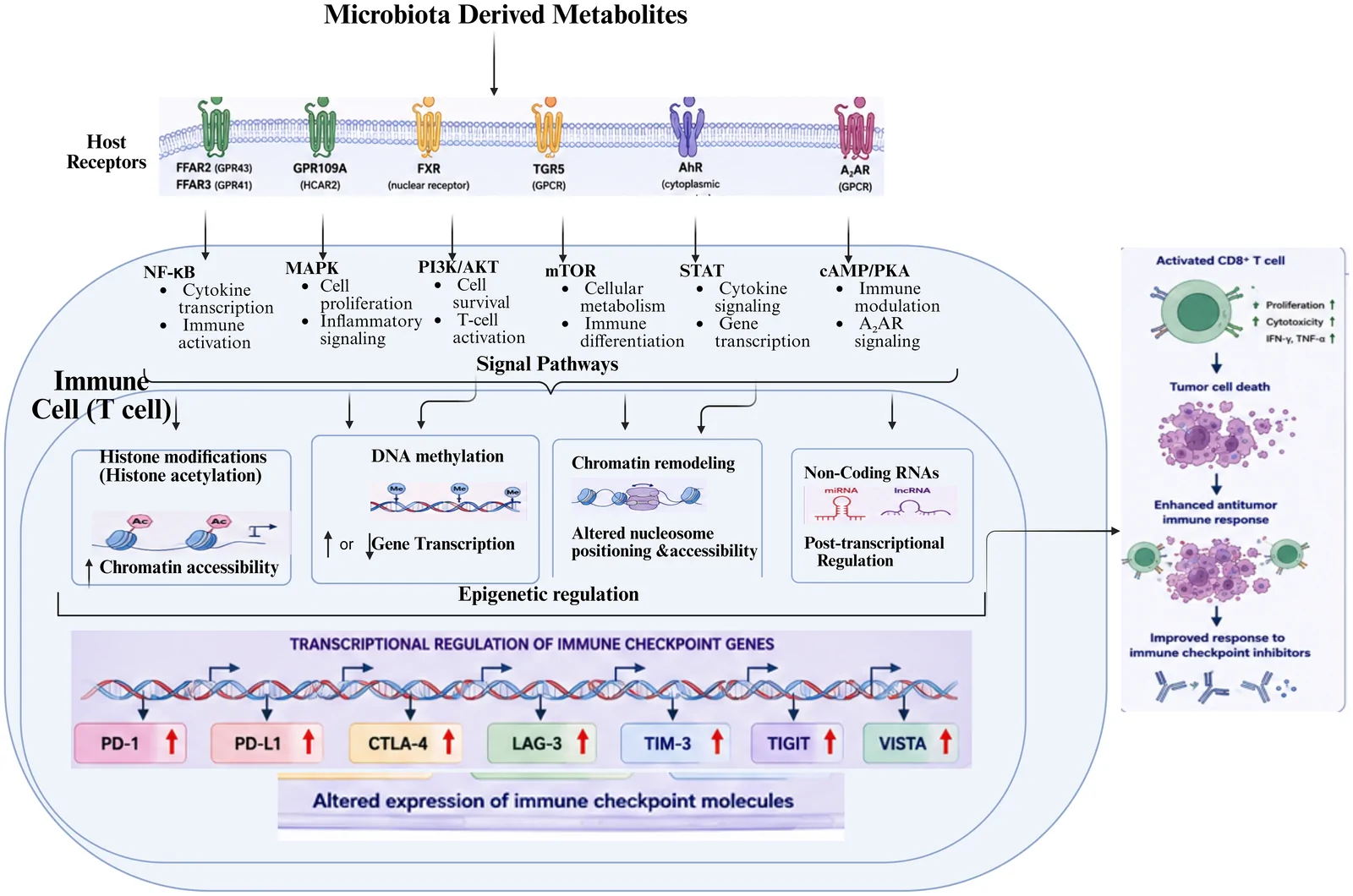
Molecular mechanisms linking microbiota-derived metabolites to immune checkpoint signaling. Microbiota-derived metabolites activate host receptors, including FFAR2/3, GPR109A, FXR, TGR5, AhR, and A_2_AR, triggering intracellular signaling pathways and epigenetic mechanisms such as histone modifications, DNA methylation, chromatin remodeling, and non-coding RNA regulation. These processes collectively modulate immune checkpoint molecules (PD-1, PD-L1, CTLA-4, LAG-3, TIM-3, TIGIT, and VISTA), thereby influencing T-cell function, antitumor immunity, and responses to immune checkpoint blockade. (Created in BioRender. Nazir, A. (2026) https://BioRender.com/pzgs1nv).

**Table 2:** Microbiota-derived metabolites and their mechanistic links to immune checkpoint regulation.

Metabolite	Main source	Molecular target/immune cells	Checkpoint link & mechanism	Evidence level	References
SCFAs	Fiber-fermenting gut bacteria	FFAR2/3, GPR109A; T cells, Tregs, DCs, macrophages	Mainly PD-1/PD-L1; GPCR/HDAC-mediated effects	Predominantly preclinical	(37, 43, 44)
Secondary bile acids	Microbial conversion of primary bile acids	FXR, TGR5; T cells, DCs, macrophages	Mainly indirect checkpoint effects through immune signaling	Primarily preclinical/indirect	(45–48)
Tryptophan-derived indoles	*Lactobacillus*, *Clostridium*, *Bacteroides*	AhR; Tregs, ILCs, DCs, macrophages	Indirect checkpoint modulation through AhR signaling	Mostly preclinical	(49–53)
Inosine	Selected gut bacteria	A_2_AR; Th1/CD8^+^ T cells	Modulates response to checkpoint blockade through T-cell activation	Strong preclinical; translational evidence emerging	(54, 66, 67)
Polyamines	Microbial amino-acid metabolism	Metabolic/epigenetic pathways; immune cells	Possible indirect checkpoint regulation	Emerging/preclinical	(54, 55)
Succinate/lactate	Microbial/host metabolism	Metabolic/hypoxia pathways; macrophages	Possible indirect effects through metabolic/inflammatory reprogramming	Emerging/preclinical	(54, 55)

### 4.1 Receptor-mediated regulation of immune checkpoint signaling

The metabolites produced by microbiota can influence immune checkpoint signaling through their action on certain receptors in the host organisms which act as sensors of microbial activity. The receptors in question are present in the intestinal epithelium and in the innate and specific immune cells whereby the microbial signals are transduced into intracellular pathways that provide for the immune response (56). Specific receptors that take part in the process are the G protein-coupled receptors (GPCR), the aryl hydrocarbon receptor (AHR), the farnesoid X receptor (FXR), the G protein-coupled bile acid receptor 1 (TGR5), and the adenosine A_2_A receptor (A_2_AR). Together those receptors are responsible for the mechanisms through which metabolites influence immune checkpoint signaling (57). Importantly, receptor activation alone does not establish direct regulation of immune checkpoints. Microbial metabolite–receptor interactions can activate downstream signaling pathways that alter transcription factor activity, cytokine production, immune-cell differentiation, or epigenetic states, which may subsequently influence checkpoint expression. Therefore, direct receptor-to-checkpoint regulation is distinguished here from indirect effects mediated through changes in immune-cell function or the surrounding immune microenvironment.

#### 4.1.1 G protein-coupled receptors

G protein-coupled receptors (GPCRs) are some of the main molecular sensors through which short-chain fatty acids (SCFAs) produced by microbiota affect host immune responses. The main SCFA-activated receptors, such as free fatty acid receptor 2 (FFAR2/GPR43), FFAR3 (GPR41), and G protein-coupled receptor 109A (GPR109A) are found in intestinal epithelial cells and various immune cell types like neutrophils, macrophages, dendritic cells, and T cells. The effect of SCFAs on these receptors initiates cell signaling pathways, which in turn allows for the regulation of cytokine secretion, immune cells migration, epithelial barrier functions, and inflammatory processes and thus play a key role in intestinal immune homeostasis (58). The activation of signal transduction by GPCRs has additionally been shown to affect the regulation of immune checkpoints through distinct signaling mechanisms, including nuclear factor (NF)-κB, mitogen-activated protein kinase (MAPK), phosphoinositide 3-kinase/protein kinase B (PI3K/AKT), and mammalian target of rapamycin (mTOR) signaling pathways among others. In the same vein, butyrate-mediated inhibition of histone deacetylases (HDACs) works in tandem with receptor-dependent signaling to provide channels for epigenetic effects that change the expression of genes relevant to immune activation and tolerance (59). Through the coordinated activation of these signaling pathways, GPCR signaling is an important player in establishing a link between microbial metabolism and immunity, including T cell activity and immune response to checkpoint inhibitors (38, 58).

#### 4.1.2 Aryl hydrocarbon receptor

The aryl hydrocarbon receptor (AhR) is a type of transcription factor that changes its shape when it binds with ligand, allowing it to act as a kind of sensor when it comes to metabolic products generated by gut microbiota and how they can influence the body’s immune system. Compounds like indole-3-acetic acid (IAA), indole-3-aldehyde (IAld), or indole-3-propionic acid (IPA) bind to AhR and cause its relocation to the cell nucleus where it has a particular control of a number of genes responsible for immune homeostasis, epithelial barrier maintenance, and responses to infections. AhR signaling determines the effectiveness of the various processes of the functioning of mucosal immunity by means of Treg differentiation and by acting as a trigger for innate lymphoid cells (ILC3) activity and stimulating interleukin-22 (IL-22) synthesis which helps to restore epithelial cells and defend against microbes (52, 60).

Although the role the AhR has in the maintenance of intestinal homeostasis is documented, it has also become clear that its signaling is an important modulator of antitumor immunity through its influence on the activation of various immune cells and the tumor immune microenvironment (61). The interactions between AhR and several transcription factors like NF-kB and STAT proteins seem to associate signals coming from microbes or inflammation with the expression and function of immune checkpoint molecules. Although the complete biochemical pathways of the influence of AhR may not be completely clarified, there is already some evidence that it serves as one of the important links between metabolism by microbes and immune checkpoints (37, 62).

#### 4.1.3 Farnesoid X receptor and G protein-coupled bile acid receptor 1

The immunomodulatory actions of secondary bile acids take place through activation of the farnesoid X receptor (FXR) and the G protein-coupled bile acid receptor 1 (TGR5) located on the intestinal epithelium, macrophages, dendritic cells, and other immune cells (63). FXR activation is responsible for the regulation of bile acid homeostasis, epithelial barrier function, and inflammation regulation, while TGR5 is dedicated to anti-inflammatory actions via cAMP-dependent pathways. Overall, these receptors link microbial bile acid metabolism to the regulation of host immunity through modulation of cytokines, immune cell differentiation, and metabolic homeostasis (47).

Besides the role in regulation of intestinal homeostasis, signaling through FXR and TGR5 is involved in shaping the tumor immune microenvironment by controlling the polarization of macrophages, maturation of dendritic cells, and functionality of T-lymphocytes. Interactions of FXR and TGR5 with signaling pathways leading to inflammation (such as NF-κB pathway) shape the activation of immune cells and indirectly contribute to immune checkpoint signaling. The interaction of bile acid receptors with immune checkpoint molecules is still being actively studied, but currently available evidence indicates that FXR and TGR5 are important molecular regulators linking bile acid metabolism by gut microbes and tumor immunity (64).

#### 4.1.4 Adenosine A_2_A receptor

The adenosine A_2_A receptor (A_2_AR) is significant for being a molecular target where the effects of inosine resulting from the microbiota can be observed. The purine metabolite inosine is obtained through the metabolism of some gut bacteria and activates the receptor A_2_AR on T cells and other immune cells. This leads to cAMP signaling pathways and the activation of several immune cells (65). In the presence of immune checkpoint blockade, the signaling pathway achieved through the receptor at A_2_AR seems to encourage Th1 cell differentiation and the secretion of interferon-γ (IFN-γ) and improve the function of CD8^+^ cells (66).

Recent studies show that the immunomodulatory effects of inosine depend on the context and are influenced by the cytokine expressions and metabolic background. By not directly regulating immune checkpoint proteins, A_2_AR signaling exerts a stimulating effect on immune activation and increases the efficacy of immune checkpoint inhibitors due to stimulation of T-cell-mediated antitumor response. These studies suggest inosine–A_2_AR signaling as a new mechanism for combining microbial metabolism with precision immunotherapy, thus warranting more studies (67).

### 4.2 Epigenetic regulation of immune checkpoint signaling

Metabolites produced by microbiota not only stimulate signaling through receptors, but also can have long-lasting effects on gene expression through the process of epigenetics. Microbiota-derived metabolites regulate the structure of chromatin and transcriptional activity without changing DNA, and therefore they influence the process of immune cell differentiation, activation, and their functions. Various mechanisms of epigenetics, for instance, modifications of histones, DNA methylation, remodeling of chromatin, and regulation of non-coding RNA, have become important elements connecting the metabolism of microbiota and immune checkpoint signaling. However, direct evidence linking specific microbiota-derived metabolites and individual epigenetic regulators to defined immune checkpoint genes remains limited, and many of these mechanisms are still based predominantly on preclinical studies. All these processes give one more level of understanding concerning how microbial metabolites manage anticancer immunity and reactions to blockade of immune checkpoints (5, 68).

#### 4.2.1 Histone modifications

One of the most researched ways that substances created by gut bacteria affect immune function is through modifications of histones. The focus is particularly on butyrate as a substance that inhibits the activity of histone deacetylases (HDACs). When the activity of HDACs is inhibited, more acetylation of histones occurs, which means there is better access of certain genes that are involved in immune responses, differentiation of cells, etc. Thus, butyrate helps with the inhibition of HDAC enzymes leading to the production of genes responsible for the anti-inflammatory processes and inhibiting excessive inflammatory signals from happening (69).

Histone acetylation has also emerged as an important mechanism that may regulate immune checkpoint signaling besides regulating overall immune response. It is well-known that histone deacetylase (HDAC) inhibition can alter chromatin accessibility thereby affecting transcription of genes that are involved in T-cell activation and tolerization as well as expression of immune checkpoint molecules such as PD-1 and PD-L1. This phenomenon may play a role in shaping the plasticity of immune cells and ensuring thus the effectiveness of immune checkpoint blockade by improving the antitumor immune response. While the exact molecular connections are being studied, numerous studies have already established roles of histone alterations as a relevant epigenetic tool for linking microbial metabolism with immune checkpoint regulation and precision immunotherapy (70).

#### 4.2.2 DNA methylation

DNA methylation is another crucial epigenetic mechanism that mediates the effects of microbiota-derived metabolites on immune regulation and signaling through immune checkpoints. This process is characterized by the addition of methyl groups to the cytosine residues of CpG dinucleotides by the action of DNA methyltransferases (DNMTs), thereby resulting in repression or activation of gene expression depending on the genetic sequence in which it is occurring. Evidence is beginning to suggest that microbial metabolites could either influence the activity of DNMTs directly or indirectly through the influence they exert on cellular metabolism and the provision of the necessary methyl donors (71).

Changes in DNA methylation may be responsible for producing abnormal levels of immune checkpoint proteins like PD-1 and PD-L1 in cancers. Microbial metabolites can change the methylation patterns of promoters of genes and may also affect pathways involved in inflammation (59, 72). While the connection between microbiota metabolites and DNA methylation has not yet been fully established, more and more data indicate that changes in DNA methylation pathways represent another pathway of the influence of the gut microbiome on antitumor immunity and immunotherapy aimed at specific targets.

#### 4.2.3 Chromatin remodeling

Chromatin remodeling is another important method of changing gene expression using microbial metabolites as it brings about changes in nucleosome position. In contrast to histone acetylation and DNA methylation, chromatin remodeling uses ATP-dependent remodeling complexes that control the accessibility of transcription factors to regulatory DNA elements. New evidence shows that microbial metabolites on this remodeling process cuts with histone-modifying enzymes so that their interaction alters immune cells’ differentiation. By changing chromatin accessibility in immune-related loci microbial metabolites are involved in transcriptional regulation of immune checkpoint molecules and functioning of immune cells. Even though this segment is still young, chromatin remodeling is another epigenetic method that connects microbial metabolism with the regulation of immune checkpoints and precision immunotherapy (73).

#### 4.2.4 Non-coding RNAs

The metabolites produced by microorganisms also have an influence on the immune response by affecting the activity of the non-coding RNA molecules - miRNAs, lncRNAs, and circRNAs. These RNAs influence post-transcriptional gene regulation via changes in mRNA stability and the processes involved in activating immune cells and inflammatory signaling. New findings suggest that microbiota metabolites are capable of changing the pattern of non-coding RNA production, which could affect certain pathways of T-cell proliferation, macrophage activation, and control of immune checkpoints. The interaction between the metabolites of the microbes and various species of non-coding RNAs is poorly understood; however, it can be concluded that these molecules form a unique link between the microbial metabolism and immunity adjustments (69). However, direct evidence linking specific microbiota-derived metabolites to non-coding RNA-mediated regulation of immune checkpoint genes remains limited. Most current evidence supports broader effects of microbial metabolites on non-coding RNA expression and immune regulation rather than a defined metabolite–non-coding RNA–checkpoint pathway. Thus, this mechanism should currently be considered an emerging area that requires further experimental validation.

### 4.3 Regulation of immune checkpoint molecules by microbial metabolites

The metabolites produced by the microbiota control immune checkpoint signaling by combining receptor-mediated signaling, epigenetic control, and metabolism reprogramming. It is important how these processes help regulate the immune checkpoint components and their functions, formation of T-cell responses, and response to tumor diseases. Understanding how microbial metabolites influence the functioning of immune checkpoint pathways will enhance the knowledge of interactions between the host and the microbiota and also facilitate the creation of therapies in the field of immunology based on the microbiome.

#### 4.3.1 Regulation of the PD-1/PD-L1 axis by microbial metabolites

The programmed cell death protein-1 and programmed death-ligand 1 (PD-1 and PD-L1) axes are the immune checkpoint pathways that have been studied the most in the context of microbiota-derived metabolites. New studies have shown that metabolite molecules of the microbiota are involved in the regulation of PD-1/PD-L1 expression through many different mechanisms that are somehow interconnected and include the processes of signaling through cell receptors and changes in cell epigenetics and metabolism. The most important metabolite in this context is represented by SCFAs but notably butyrate which is known to regulate PD-1/PD-L1 expression due to the inhibition of histone deacetylases and resultant opening of chromatin and modification of the way immune checkpoint vital genes are expressed. It needs to be noted that changes that occur at the epigenetic level affect signaling in T-cells and lead to improvements in immune tolerance and change the mode of operation of the tumor immune microenvironment (74, 75). However, much of this evidence is preclinical, and the effects may vary according to the immune-cell type and experimental context; therefore, direct regulation of PD-1/PD-L1 by butyrate requires further validation.

Besides SCFAs, secondary bile acids, metabolites derived from tryptophan, and inosine also have indirect effects on the PD-1/PD-L1 pathway, which they can regulate by regulating the processes of inflammatory signaling, cytokine production, and differentiation of immune cells via receptors such as FXR, TGR5, AhR, and A_2_AR. The coordinated signaling pathways affect the activities of dendritic cells, macrophages, and cytotoxic CD8^+^ T cells, thereby affecting the efficiency of immune checkpoint blockade therapy. Clinical and preclinical research demonstrates that the variations in gut microbiota composition and their metabolites that are produced affect the efficiency of immune checkpoint blockade therapy as well, which shows that microbial metabolites can serve as therapeutic adjuvants for immunotherapy and act as predictive biomarkers in immunotherapy.

#### 4.3.2 Regulation of CTLA-4 and emerging immune checkpoint molecules

Recent findings show the relationship between microbial metabolites enzymes and such immune checkpoint molecules like cytotoxic T-lymphocyte-associated protein 4 (CTLA-4), lymphocyte activation gene-3 (LAG-3), T immune globulin and mucin-domain containing-3 (TIM-3), T-cell-immunoglobulin and ITIM domains (TIGIT), and V-domain Ig suppressor of T cell activation (VISTA). Although information about their functioning mechanisms remains unexplored compared to PD-1/PD-L1, it is presumed that microbial metabolites affect these checkpoints via some kinds of receptor-mediated signaling, epigenetic remodeling, and a changing role of inflammatory cytokines which consequently influences T-cell activation as well as immune tolerance (64, 76).

The different short-chain fatty acids, bile acids generated from primary, and metabolites which are generated from tryptophan influence the maturation and function of Tregs, dendritic cells, and macrophages acting indirectly on many immune checkpoints. Moreover, the effect of microbial metabolites on chromatin accessibility and DNA methylation leads to other possible ways of the immune checkpoints’ regulation. However, compared with the PD-1/PD-L1 axis, evidence for direct regulation of CTLA-4, LAG-3, TIM-3, TIGIT, and VISTA by specific microbial metabolites remains limited and is largely based on preclinical or indirect observations. However, there is a reasonable necessity to conduct further studies for better understanding of this issue (13, 17).

#### 4.3.3 Clinical relevance of microbial metabolite–immune checkpoint interactions

The increasing knowledge of the relationship between metabolites coming from microbiota and immune checkpoints has implications for immunotherapy and precision medicine in combating cancer. The changes in immune signalling, caused by receptor pathways, epigenetic mechanisms, and gene therapy, due to microbial metabolites determine the effectiveness and durability of immune checkpoint blockade treatment. Moreover, changes in gut microbiome composition and the metabolite production were connected with differences in clinical outcomes, resistance towards treatment, and adverse effects related to immunologic factors, which indicates the potential role of microbial metabolites as predictive biomarkers and treatment targets. Thus, it can be concluded that the obtained data prove the efficacy of utilizing microbiome-based approaches together with immune checkpoint inhibitors for increasing patient management (37, 43, 66).

## 5 Therapeutic implications of the metabolite–immune checkpoint axis

The increasing realization about the interactions between microbial-derived metabolites and immune checkpoints has opened new avenues for immunotherapy in oncology. Strategies based on microbiome manipulation, including the alteration of microbiota composition and the production of metabolites, are gradually being positioned as the most favorable opportunities to enhance the process of immune checkpoint blockage.

### 5.1 Microbiome-based therapeutic strategies

The therapeutic strategies discussed below are supported by varying levels of evidence and should therefore be interpreted according to their stage of development. While several microbiome-modulating approaches have shown promising effects *in vitro* and in preclinical models, clinical evidence for their ability to consistently modify immune checkpoint responses remains limited. Probiotics, postbiotics, FMT, and engineered microbial approaches should therefore be considered investigational in this context unless supported by clinical studies. The purpose of microbiome-focused therapies is to boost the effectiveness of immune checkpoint inhibitors via altering gut bacteria composition and metabolism. Diet, prebiotics, probiotics, postbiotics, fecal microbiota transplant (FMT) and engineered microorganisms can be considered as promising methods of restoring the balance of microbiome organisms and producing essential metabolites to enhance efficacy. Such methods stimulate immune cell activity, improve the effectiveness of epithelial barrier, and therefore improve the immune environment in tumors and provide better results in anti-tumor immunity (77, 78).

Early clinical studies of FMT in patients with cancer receiving immune checkpoint inhibitors have reported encouraging findings; however, further clinical validation is required. Likewise, it is also explored the potential safety and consistency of the use of postbiotics and microbiota metabolites like SCFA and tryptophan metabolites instead of live microbials due to their composition pattern and immune modulating properties. Also, advances in synthetic biology yield the possibility of developing engineered microbes that could deliver therapeutic metabolites or immune-modulating compounds directly into the intestine (37, 79).

### 5.2 Precision medicine and biomarker development

The progress made in the field of microbiome research indicates that metabolites produced by microbiota holds great promise for being used as biomarkers to help in the prediction of responses to immune checkpoint inhibitors and the recommendation of personalized immunotherapy. It has been noted that some differences in microbial composition and metabolites may indicate that treatment efficacy, immune-related adverse events, and resistance to immune checkpoint blockade may vary. The application of multi-omics strategies, which can combine metabolomics and genomic, transcriptomic, and immunological data, can enhance the possibility of better patient stratification and identification of those who are more likely to benefit from interventions aiming at influencing the microbiome (80).

To enhance the precision of immunotherapy, it is also necessary to conduct standardized evaluation of biomarkers, sophisticated analytical platforms, and clinical studies to define the predictive significance of metabolites generated by the microbiota. Progress in the areas of artificial intelligence, machine learning, and systems biology will continue to facilitate the discovery of biomarkers, maximize the effectiveness of immunotherapy, and create personalized microbiome-based therapies minimizing side effects (81).

### 5.3 Challenges and future perspectives

Although many strides have been made regarding our understanding of metabolic processes involving immunology and gut microbiota, translating this knowledge into clinical settings poses many remaining challenges. Interindividual differences between gut microbiota composition, effects of diet, the variability of illnesses that different patients suffer from, and lack of standardized protocols that may facilitate the understanding of varied conclusions that previous studies may indicate all make it difficult to achieve consistent results in studies. Further complicating the issue is our understanding of the relationships between microbial metabolites, immunity, and tumor microenvironment, which is still incomplete.

Future studies should focus on applying various cutting-edge technologies, including multi-omics and artificial intelligence, for the purpose of target selection, and clinical application for developing effective microbiome-based therapies. The collaborative efforts of microbiologists, immunologists, oncologists, and computational scientists are expected to facilitate therapeutics targeting human microbiome. Interpretation of microbiome–immunotherapy associations is further complicated by potential confounding factors, including diet, antibiotic exposure, cancer type, treatment regimen, geography, age, sex, and host genetics, all of which may influence microbiota composition and therapeutic response. The various microbiome-based therapeutic approaches have been categorized on the basis on their mechanism of action, clinical significance, and current stages of development; the findings have been compiled in **Table 3**.

**Table 3:** Microbiota-derived metabolites, principal receptors, mechanisms of immune checkpoint regulation, and therapeutic applications.

Therapeutic strategy	Mechanism of action	Primary target/metabolites	Potential clinical benefit	Current status	Representative references
Dietary interventions	Increase production of beneficial microbial metabolites through high-fiber diets	SCFAs	Enhance antitumor immunity and improve responses to immune checkpoint inhibitors	Clinical/Preclinical	(36, 43)
Prebiotics	Promote growth of beneficial gut bacteria	SCFAs, indoles	Restore microbial homeostasis and modulate immune responses	Clinical	(65, 78)
Probiotics	Restore beneficial microbial communities	Multiple microbial metabolites	Improve immune function and support immunotherapy	Clinical	(37, 65)
Postbiotics	Direct administration of microbial metabolites or bioactive products	SCFAs, indole metabolites	Standardized immune modulation without live microorganisms	Preclinical/Early clinical	(37, 79)
Fecal microbiota transplantation (FMT)	Restores microbiome diversity from healthy donors	Multiple metabolites	Overcome resistance to immune checkpoint inhibitors	Clinical	(66, 78)
Engineered microbial therapeutics	Targeted delivery of therapeutic metabolites or immunomodulators	SCFAs, inosine, engineered metabolites	Precision microbiome-based immunotherapy	Experimental	(79, 80)

## 6 Conclusion

The metabolites produced by the microorganisms in the microbiota have been recognized as significant molecules connecting the gut microbiota to the regulation of the immune system and the signaling in immune checkpoints. With the assistance of participated signaling through the receptors, epigenetic regulation, and metabolic reprogramming, these metabolites regulate the expression of numerous critical immune checkpoint molecules (PD-1, PD-L1, CTLA-4, LAG-3, TIM-3, TIGIT, and VISTA) influencing the processes of immune homeostasis, antitumor response, and responses to treatment. This review provides evidence supporting the Metabolite-Immune Checkpoint Axis as a good framework to explain how microbial metabolism affects the process of immune regulation and impacts the outcomes of various diseases (56, 82). The advancements in microbiome research, metabolomics, and immunology have opened up new approaches to the use of microbial metabolites for targeted immunotherapy. Approaches involving microbiome interventions, including changes in diet, prebiotics, probiotics, postbiotics, fecal microbiota transplantation, and engineered microbial interventions, are viewed as promising practices that may improve immune checkpoint blockade and clinical outcomes.

Nevertheless, several obstacles remain, including the heterogeneity of the microbiome composition between individuals, lack of mechanisms understanding, the need to standardize analytical approaches, and proper validation of clinical studies. Therefore, future research in the area is likely to benefit from the integration of multi-omics technologies, artificial intelligence, and well-thought-out clinical trials to find relevant biomarkers and develop personalized therapies based on the microbiome. Research aiming to study the metabolites produced by the microbiota would increase our understanding of host-microbiota interactions and make it possible to put these mechanisms into practice.
